# Lewis-A Antibody in Clinical Practice: A Case Report

**DOI:** 10.7759/cureus.64501

**Published:** 2024-07-13

**Authors:** Arthi R, Soundharya V, Hari Haran A, Suresh K I, Sahayaraj James

**Affiliations:** 1 Transfusion Medicine, Saveetha Medical College and Hospital, Saveetha Institute of Medical and Technical Sciences, Chennai, IND

**Keywords:** anti-lewis a, blood group discrepency, naturally occurring antibody, antibody identification, alloimmunization

## Abstract

Anti-Lewis antibodies are often not clinically significant since they do not react at 37°C. These antibodies have, however, occasionally been linked to hemolytic transfusion reactions (HTR). We report a case of naturally occurring anti-Lewis-a (Le-a) in a 58-year-old patient found during routine blood grouping. As Lewis antigen is a low-prevalence antigen, compatible units were found after crossmatching two units of packed red cells. Lewis blood group antigen antibodies frequently react at lower temperatures and remain clinically insignificant, but in rare cases, they may react at a higher temperature of 37°C and cause a hemolytic episode or impair the lifespan of incompatible red blood cells in the recipient. Hence, antigen-negative crossmatch compatible units should be used for transfusion. In an emergency, the donor's register, with its comprehensive phenotypic profile, can be quite helpful in supplying blood for transfusions.

## Introduction

In 1946, Mourant discovered the antibody anti-Lewis-a (Le-a) [1]. The antithetical antibody was discovered by Anderson in 1948. Grubb proved that saliva and plasma contained soluble Lewis antigens in 1951 [2].

Le-a and Lewis-b (Le-b) antigens are not synthesized on red blood cells (RBC) and are adsorbed from plasma. Besides being present in secretions and RBCs, Lewis antigens are also found on platelets, endothelium, kidney, genitourinary and gastrointestinal epithelium [3]. Their synthesis is linked to the secretor (Se) gene (FUT2) and Le gene (FUT3) on chromosome 19p13.3 and 19q13.3, respectively. The Le gene produces a fucosyl transferase enzyme, adding fucose to the type I precursor chain to create Le-a. With the presence of Se and Le genes, Le-b antigen forms by adding fucose to the type 1 H-chain. Notably, Le (a-b+) individuals are secretors, while Le (a+b-) individuals are non-secretors, as red cells, utilizing type 2 chains, adsorb Lewis antigens from plasma onto their membrane [2].

Lewis antibodies were formerly believed to have a minimal impact on clinical transfusion practice; however, new data indicate that these antibodies have become increasingly significant in the context of transplants and transfusions [4]. These antibodies may agglutinate and cause complement-mediated hemolysis, showing clinical significance even if not reactive at 37°C [5]. Interestingly, there is evidence of anti-Lewis antibodies resulting in hemolytic transfusion reactions [5]. We report a case of naturally occurring Le-a antibody in clinical practice.

## Case presentation

A 58-year-old male patient had come to the outpatient department with a complaint of abdominal pain, pedal edema with rashes, and swelling of both lower limbs. He was diagnosed as a case of hypertensive urgency and acute gastritis with infected scabies. He was a known case of systemic hypertension and type 2 diabetes mellitus for the past three years but was on irregular medication. He was also a chronic alcoholic. The patient had a history of cholecystectomy with common bile duct exploration with T-tube insertion done three years back. He had no history of transfusions or transplantations.

**Figure 1:**
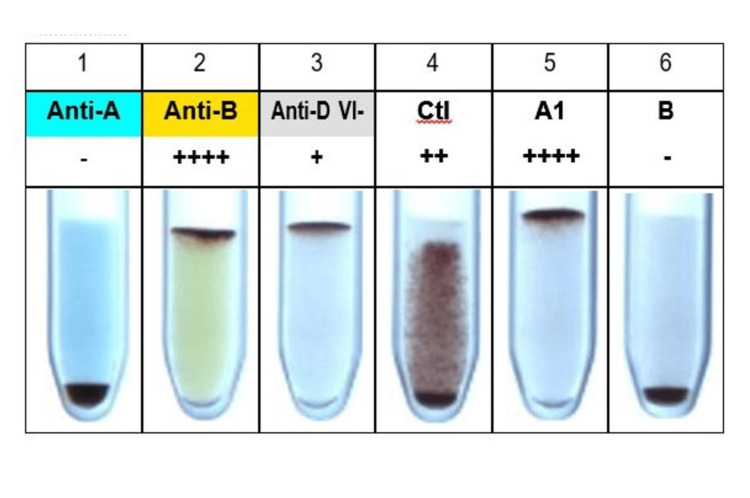
Forward and reverse blood grouping by Bio-Rad DiaClon

Routine investigations were sent, including an ethylenediamine tetraacetic acid (EDTA) sample, to our blood center for blood grouping and typing. Blood grouping and typing were done by column agglutination using Bio‑Rad DiaClon (Bio-Rad Laboratories, Hercules, California), which gave the result shown in Figure 1.

**Figure 2:**
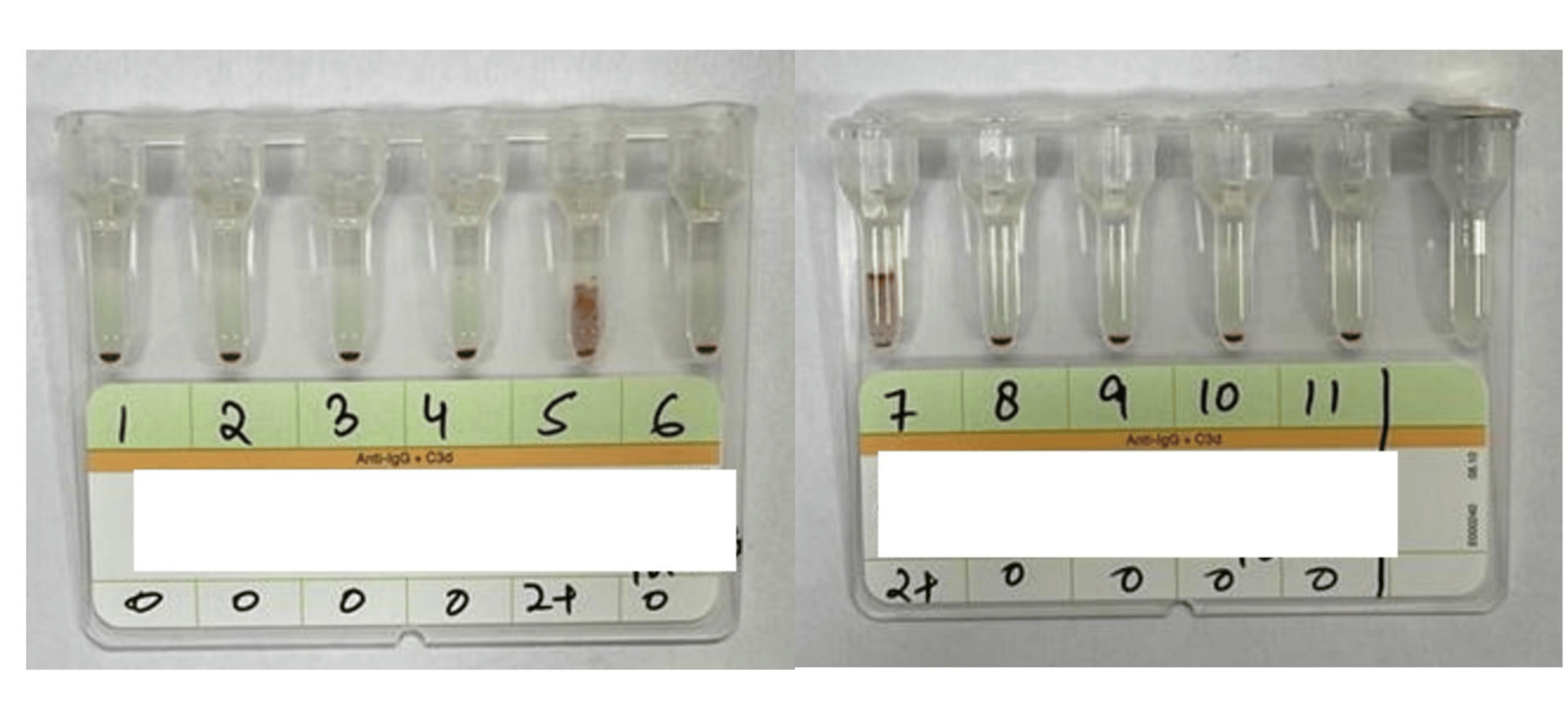
Antibody identification cell panel (Bio-Rad ID-Dia 11-cell panel) showing the cell reactivity pattern

Forward grouping showed B Rh positive. Reverse grouping showed 2+ positivity with O cells. There was a discrepancy between the forward and reverse grouping, suggesting a group IV grouping discrepancy. To further evaluate the excess antibodies, a serum sample was requested. Indirect Coomb's test showed 2+ positivity. Antibody screening and identification were done using Bio‑Rad ID-DiaCell I, II, III Asia (Mia+), a three-cell panel (lot number 907084.56), and Bio‑Rad ID-Dia 11-cell panel (lot number 45161.73), respectively. The three-cell panel showed 2+ reactivity in the second cell line. The results of the antibody identification with the Bio‑Rad ID-Dia 11-cell panel are shown in Figure 2.

Bio‑Rad ID-Dia 11-cell panel showed 2+ reactivity in 5 and 7 cell lines. After elucidation by the antigram provided by the manufacturer, Lewis-a was identified in the patient's serum. Phenotyping of the patient's RBC was done, and it was found to be Le (a-b‑).

To check the secretor status, a saliva sample was collected from the patient and processed. The secretor status was determined by the hemagglutination inhibition test. An inhibition test with Lewis antisera showed that he was a secretor.

The patient was planned for debridement and required reservation of one unit of packed red blood cells before the procedure. The crossmatching procedure was done using Bio‑Rad column agglutination gel card (Anti‑IgG + Anti‑C3d cards). Among the two bags crossmatched, one was found to be incompatible, and the other was compatible. However, the patient did not require any blood transfusion during his hospital course. 

## Discussion

Lewis antibodies are naturally occurring, mostly IgM antibodies that have minimal clinical significance for the following reasons [3]. First of all, Lewis antigens are soluble, which allows the red blood cells from the donor to lose their own antigens. Second, the recipient antibodies frequently neutralize these released antigens. Thirdly, there is a clear preference for these antibodies to react at lower temperatures. Fourth, non-O group individuals express Lewis antigens at a lower level than O group individuals, which means that crossmatch incompatibility is infrequent. Lastly, the majority of Lewis antibodies are naturally occurring IgM antibodies; reports of the IgG class have only been made in rare instances [6] [7].

Of the Lewis antibodies, anti-Le-a is the most commonly found. The most common Lewis antibody, anti-Le-a, is frequently found in room temperature testing and occasionally reacts at both indirect antiglobulin test and at 37°C [8]. Anti-Le-b, which can bind complement, is not as prevalent or strong as anti-Le-a [9]. Anti-Le-a antibodies are more commonly linked to acute hemolytic transfusion reactions (HTR) than anti-Le-b antibodies. There have also been reports of delayed HTR cases [10].

Antibodies against Lewis are frequently detected in the sera of pregnant women and in those with the Le (a-b-) phenotype [11]. Fetal cells express Lewis antigens poorly; hence, the likelihood of hemolytic disease in a fetus and newborn is quite low. In a study by Rajeshwari et al., Lewis antibodies were most commonly found in pregnant women, all with the Le (a-b-) phenotype [3]. The prevalence of Le-a and Le-b antigens in Indian blood donors is 17.4% and 45.6% respectively [12]. Hence, it is not very difficult to find antigen-negative units for transfusion.

Lewis antibodies are not frequently linked to HTRs because most Lewis antibodies are not thought to be active at 37°C, and the Lewis antigens in the transfused blood's plasma may neutralize the recipient's Lewis antibodies [11]. However, despite negative pre-transfusion sample testing, patients with anti-Lewis antibodies have been linked to acute HTRs [13]. This might be because of two reasons. First, Lewis antigens that are soluble in the donor's blood plasma may obscure the presence of clinically significant anti-Lewis antibodies. It is likely possible to avoid false negative results and acute hemolytic transfusion reactions by washing donor red blood cells prior to a crossmatch at 37°C with anti-human globulin (AHG). Second, variations in plasma volume (e.g., pregnancy), hematocrit (e.g., hemolysis), and lipid levels (e.g., liver illness) cause physiological fluctuations in the concentration of anti-Lewis antibodies and Lewis antigens [7]. This may also result in the undetectable state of clinically relevant anti-Lewis antibody titers at any particular time during pre-transfusion testing.

Transplant medicine is another field of medicine where anti-Lewis antibodies have been considered to be clinically irrelevant. On the other hand, Le (a-b-) phenotypic recipients have experienced kidney allograft rejections as a result of donor Lewis antigen incompatibility [14]. Spitalnik et al. noted that recipients of Lewis incompatible kidney allografts having anti-Le-a or anti-Le-b antibodies later showed signs of a complete rejection of allografts [15].

## Conclusions

Anti-Lewis antibodies are uncommon and often evade detection, making it challenging to investigate their clinical significance. Consequently, whenever clinically significant anti-Lewis antibodies are found during a patient's lifetime, it may be beneficial to provide Le-a and Le-b antigen-negative red blood cell units crossmatched at 37°C with pre-washed donor red blood cells and AHG. This would prevent the development of clinically significant anti-Le-a and anti-Le-b antibodies in individuals with the Le (a-b-) phenotype and decrease false negative crossmatch results, particularly for those receiving chronic red blood cell transfusions.
